# C–X–C motif chemokine ligand 12: a potential therapeutic target in Duchenne muscular dystrophy

**DOI:** 10.1080/21655979.2021.1967029

**Published:** 2021-08-23

**Authors:** Jie Chen, Xinsheng Lai

**Affiliations:** a School of Life Science, Nanchang University, Nanchang, Jiangxi, China; b Laboratory of Synaptic Development and Plasticity, Institute of Life Science, Nanchang University, Nanchang, Jiangxi, China

**Keywords:** Duchenne muscular dystrophy, bioinformatic analysis, differentially expressed genes, *cxcl12*, glucocorticoids

## Abstract

Duchenne muscular dystrophy (DMD) is an X-linked recessive disease caused by a mutant dystrophin protein. DMD patients undergo gradual progressive paralysis until death. Chronic glucocorticoid therapy remains one of the main treatments for DMD, despite the significant side effects. However, its mechanisms of action remain largely unknown. We used bioinformatics tools to identify pathogenic genes involved in DMD and glucocorticoid target genes. Two gene expression profiles containing data from DMD patients and healthy controls (GSE38417 and GSE109178) were downloaded for further analysis. Differentially expressed genes (DEGs) between DMD patients and controls were identified using GEO2R, and glucocorticoid target genes were predicted from the Pharmacogenetics and Pharmacogenomics Knowledge Base. Surprisingly, only one gene, *CXCL12* (C–X–C motif chemokine ligand 12), was both a glucocorticoid target and a DEG. Kyoto Encyclopedia of Genes and Genomes pathway enrichment analysis, Gene Ontology term enrichment analysis, and gene set enrichment analysis were performed. A protein–protein interaction network was constructed and hub genes identified using the Search Tool for the Retrieval of Interacting Genes (STRING) database and Cytoscape. Enriched pathways involving the DEGs, including *CXCL12*, were associated with the immune response and inflammation. Levels of CXCL12 and its receptor CXCR4 (C–X–C motif chemokine receptor 4) were increased in X-linked muscular dystrophy (mdx) mice (DMD models) but became significantly reduced after prednisone treatment. Metformin also reduced the expression of *CXCL12* and *CXCR4* in mdx mice. In conclusion, the CXCL12–CXCR4 pathway may be a potential target for DMD therapy.

## Introduction

Duchenne muscular dystrophy (DMD) is an X-linked recessive neuromuscular disorder characterized by progressive muscle weakness and wasting [1,2] that affects 1 in every 3500–5000 boys [3]. The clinical symptoms of DMD patients usually start at the age of 3 to 5 years and progress quickly [4]. In the absence of treatment, the patients become completely incapacitated by the age of 13 to 15 years, followed by myocardiopathy and respiratory failure that ultimately lead to death in the third decade of life [5–7].

DMD is caused by mutations in the gene encoding dystrophin, a stabilizing protein that forms a bridge between the actin cytoskeleton and the sarcolemma of muscle fibers. In DMD, a lack of dystrophin disrupts this bridge and breaks down the membrane integrity of muscle fibers, leading to muscle wasting and degeneration [8]. Currently, there is no cure for DMD. However, the positive effect of chronic glucocorticoid treatment is well supported, and this treatment is currently recommended as the standard of care for DMD [5,9,10]. Glucocorticoid therapy in DMD can effectively improve muscle strength or function and delay the loss of ambulation [11]. Recently, the glucocorticoid deflazacort has been approved for DMD treatment by the US Food and Drug Administration (FDA). However, the molecular mechanisms by which glucocorticoids act in the treatment of DMD are largely unknown. Moreover, glucocorticoid treatment risks significant side effects, including growth disorders, obesity, osteoporosis, fractures, cataracts, and adrenal insufficiency [12]. Exploring new drugs that can be used in combination therapy with glucocorticoids may be helpful for the prognosis of patients with DMD. To facilitate the search for molecules that can achieve similar therapeutic effects, there is a need for deeper insight into the major cellular pathways affected by synthetic glucocorticoid administration in DMD.

Gene microarray analysis has been extensively applied not only in the identification of differentially expressed genes (DEGs) between patients and healthy controls, but also in clinical biomarker screening, drug development, and prediction of signaling pathways involved in diseases [13–17]. In the present study, two gene expression profile datasets (GSE38417 and GSE109178) containing data from 34 DMD patients and 12 healthy controls were downloaded from the Gene Expression Omnibus (GEO). Samples were screened for DEGs, and 217 genes were found to be downregulated, whereas 1170 were upregulated. The Pharmacogenetics and Pharmacogenomics Knowledge Base (PharmGKB) was used as a reference to identify genes targeted by glucocorticoids and determine which of them were also among the DEGs in our sample. Subsequently, Gene Ontology (GO) and Kyoto Encyclopedia of Genes and Genomes (KEGG) pathway enrichment analyses were conducted using the DEGs. A protein–protein interaction (PPI) network was visualized based on the products of the DEGs, and the genes encoding the top 10 proteins with the most interactions were selected for further examination; these included the glucocorticoid target gene *CXCL12* (C–X–C motif chemokine ligand 12), which was ranked eighth. Next, gene set enrichment analysis was performed using *CXCL12*. We verified that the mRNA expression levels of *CXCL12* and its receptor *CXCR4* were higher in mdx mice than in wild-type (WT) mice. Moreover, using the GSE95682 dataset from GEO, we found that *CXCL12* levels were decreased in mdx mice after treatment with prednisone. We also found declines in *CXCL12* and *CXCR4* levels after 1 – and 2-month periods of treatment with metformin, a potential therapeutic drug for DMD. In conclusion, we used bioinformatics to identify and analyze *CXCL12* as a possible new target for the treatment of DMD. Further study of this gene may facilitate the development of DMD drugs with fewer side effects. Our findings provide insight into the molecular mechanisms involved in glucocorticoid treatment of DMD and may aid in the identification of novel targets for DMD drugs, as well as novel candidate biomarkers for DMD diagnosis and prognosis.

## Materials and methods

### Identification of glucocorticoid target genes

Gene targets of glucocorticoid drugs were identified using the Pharmacogenetics and Pharmacogenomics Knowledge Base (PharmGKB) database (https://www.pharmgkb.org), which is a repository of information on the relationships between drugs and genes [18,19].

### Microarray data

Microarray gene expression datasets (GSE38417 and GSE109178) were downloaded from the GEO database of the National Center for Biotechnology Information (https://www.ncbi.nlm.nih.gov/) [20,21]. The platform for both GSE38417 and GSE109178 is GPL570 (Affymetrix Human Genome U133 Plus 2.0 Array).

### Identification of DEGs

DEGs were compared between human muscle tissues from DMD patients and healthy controls using GEO2R (https://www.ncbi.nlm.nih.gov/geo/geo2), which is an interactive web tool that allows users to screen for DEGs by comparing more than two datasets in a GEO series [22]. Differences with an adjusted *p*-value of <0.01 and a |log2 FoldChange| value of ≥1.5 were considered statistically significant. A Venn diagram tool (http://bioinformatics.psb.ugent.be/webtools/Venn/) was used to identify DEGs common to both GSE38417 and GSE109178, and these common DEGs were further screened to identify which of them were glucocorticoid target genes.

### GO functional enrichment analysis and KEGG pathway enrichment analysis

The Gene Functional Classification Tool of the DAVID online bioinformatics database (https://david.ncifcrf.gov/) can be used to classify functionally related genes into one group and explain their role in the biological environment [23]. KEGG is a comprehensive database that integrates genomic, chemical, and system functional information [24,25]. The GO database can be used to analyze a biological process in terms of its function, the biological pathways involved, and its localization in cells [26]. In this study, the DAVID database was used to analyze the functions of the DEGs. Statistical significance was set at *p* < 0.05.

### PPI network analysis

A PPI network was constructed by identifying interactions between proteins using the Search Tool for the Retrieval of Interacting Genes (STRING) database (https://string-db.org/) [27] and visualizing them in Cytoscape (version 3.8.2), an open-source web visualization and analysis software [28]. The multiscale curvature classification (MCC) algorithm of the cytoHubba app was used to identify the top 10 genes.

### Gene set enrichment analysis

The GSEA analysis (version 4.1.0) was used to perform the influence of target gene expression on biochemical pathways [29]. Target genes were divided into two groups, high-expression (top 50% in strength of expression) and low-expression (bottom 50%).

### Mouse lines and drug treatment dose

Muscular dystrophy model (mdx) mice (C57BL/10ScSn-Dmdmdx/J) were purchased from the Jackson Laboratory (Bar Harbor, ME, USA; stock #001801). Twenty mdx mice and 10 WT (C57) mice aged ~5 weeks were acclimatized for 1 week. Six mdx mice were randomly selected and divided into two groups. One group received daily intraperitoneal injections of metformin (D150959-5 G, Sigma, St. Louis, MO, USA; 200 mg/kg; n = 3), and the other group received daily injections of normal saline (0.9% NaCl; n = 3). All six mice received injections for 30 days. Another two groups of mdx mice received injections for 60 days under the same conditions and methods. The experimental procedures were approved by the Institutional Animal Care and Use Committee of Nanchang University.

### RNA extraction and quantitative real-time PCR (qRT-PCR) analysis

Total RNA was isolated from the gastrocnemius using Trizol reagent (Invitrogen, Grand Island, NY, USA), and cDNA was converted from RNA using a reverse transcription kit (Takara, Shiga, Japan) and oligo(dT) primers. qRT-PCR was performed using SYBR GreenER qPCR mix (Thermo Fisher, Waltham, MA, USA) with gene-specific primers. The primer pairs were the following: *CXCL12* forward, 5′-GCTGTGCATCTACACCGACA-3′; *CXCL12* reverse, 5′-AGTGAGGATGGAGACCGTGGTG-3′; *CXCR4* forward, 5′-GACTGGCATAGTCGGCAATGGA-3′, *CXCR4* reverse, 5′-CAAAGAGGAGGTCAGCCACTGA-3′. GAPDH was used as a reference for each sample.

### Western blot

Western blotting was performed as previously described [30]. Briefly, protein samples were separated by SDS-PAGE (sodium dodecyl sulfate polyacrylamide gel electrophoresis) and transferred to a nitrocellulose membrane. The membrane was blocked in 5% nonfat milk for 1.5 h at room temperature and incubated with primary antibodies overnight at 4°C: anti-CXCL12 (1:2000; Sangon, Shanghai, China), anti-CXCR4 (1:3000; Sangon), and anti-GAPDH (1:2000; Abcam, Cambridge, UK). The membranes were then incubated with HRP-conjugated secondary antibodies (1:2000; Invitrogen, Waltham, MA, USA) for 2 h at room temperature. Immunoreactive protein bands were visualized using an HRP chemiluminescence detection reagent (ECL, Thermo Fisher), and blots were imaged using a ChemiDoc MP imaging system (Bio-Rad, Hercules, CA, USA).

## Results

Chronic glucocorticoid therapy remains one of the main treatments for DMD. However, the molecular mechanisms by which glucocorticoids act in the treatment of DMD are largely unknown. The PharmGKB database was used to identify target genes of the glucocorticoids prednisone and prednisolone, which were then cross-referenced with the DEGs found in both of the two GEO datasets (GSE38417 and GSE109178); this process resulted in the identification of *CXCL12* as both a glucocorticoid target and a DEG. Next, GO analysis, KEGG analysis, gene set enrichment analysis, and PPI analysis were used to identify the major signaling pathways involved in DMD. Subsequently, we verified that the mdx mouse model of DMD showed increased levels of CXCL12 protein and its receptor CXCR4, as well as increased levels of *CXCL12* and *CXCR4* mRNA. However, after treatment with metformin and prednisone, expression of CXCL12 and CXCR4 in the DMD model mice was reduced, suggesting that *CXCL12* may be a potential therapeutic target for DMD.

### Glucocorticoid target genes

Prednisone and prednisolone are the two glucocorticoids most commonly used as drugs. To investigate the mechanism of action of glucocorticoids in DMD treatment, we began by predicting potential target genes of prednisone and prednisolone. We searched for ‘prednisone and prednisolone’ in the PharmGKB database, and 12 potential target genes were identified, as shown in Table 1.

**Table 1.** Targets gene of the prednisone and prednisolone

Drug	Gene	Variant
prednisone	CXCL12	rs1801157
	DROSHA	rs639174
	DOK5	rs117532069
	ABCB1	rs104562, rs229109
	GSTA1	rs3957357
	CTLA4	rs4553808
	LINC00251	rs141059755
	BMP7	rs79085477
	GATA3	rs3824662
prednisolone		
	ABCB1	rs104562, rs229109
	DSE, TSPYL1	rs3828743
	PANPLA	rs738409

### Identification of DEGs between DMD and healthy control

**Figure 1.**
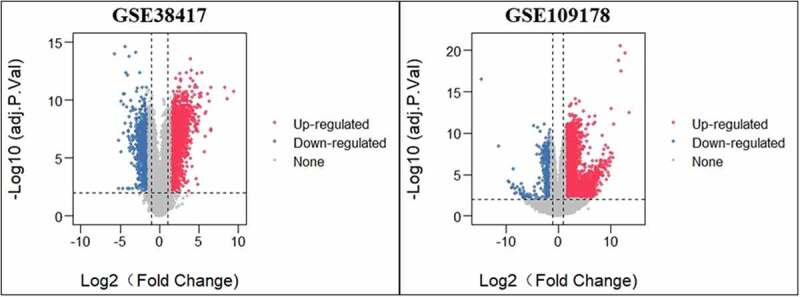
Volcano plots of DEGs from GSE38417 and GSE109178

**Figure 2.**
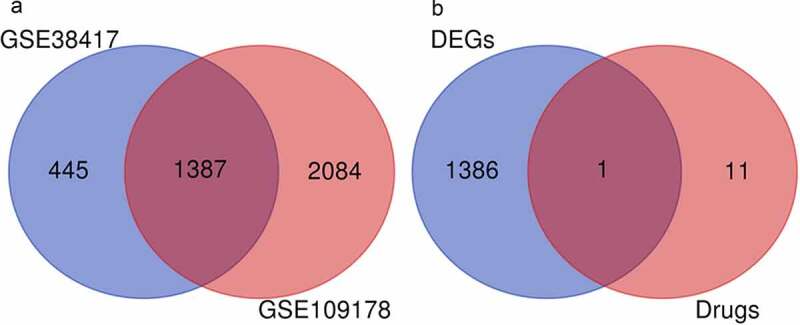
**Identified DEGS and their overlap with glucocorticoid target genes**. (a) Venn diagram of DEGs common to the GSE38417 and GSE109178 datasets. (b) Venn diagram of DEGs and glucocorticoid (prednisone/prednisolone) target genes

To identify genes involved in DMD, the GEO database was searched for gene expression profiles that included data from human muscle samples taken from DMD patients, had a sample size ≥5, and were published in the last 10 years. Two gene expression profiles (GSE38417 and GSE109178) met the criteria and were downloaded for further analysis. After standardizing these two microarray datasets, we identified the DEGs between DMD patients and healthy controls. As shown in Figures 1, 2379 DEGs (614 downregulated and 1764 upregulated) were found in GSE38417 (16 DMD patients and 6 healthy controls), and 4451 DEGs (446 downregulated and 4405 upregulated) were observed in GSE109178 (17 DMD patients and 6 healthy controls); differences were considered statistically significant at an adjusted *p*-value of <0.01 and a |log2FoldChange| value of ≥1.5. Between the two GEO series, there were 1387 genes that were DEGs in both (217 downregulated and 1170 upregulated), as shown in the Venn diagram in Figure 2a. When we checked the overlap between these DEGS and the 12 predicted glucocorticoid target genes, we found only one gene belonging to both sets: *CXCL12* (GSE38417: logFC = 1.9, *p* < 0.001; GSE109178: logFC = 1.7, *p* < 0.001), shown in Figure 2b.

### GO functional enrichment analysis and KEGG pathway enrichment analysis

**Figure 3.**
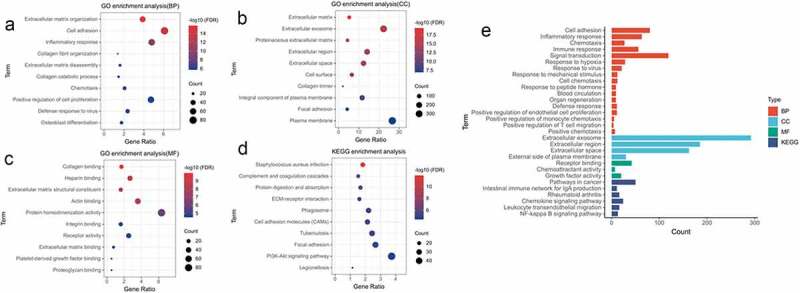
**GO and KEGG enrichment analyses of DEGs**. (a) biological process (BP). (b) cell composition(CC). (c) molecular function(MF). (d) KEGG pathway enrichment analysis. (e) BP, CC, MF and KEGG enrichment analysis of pathways containing *CXCL12.*

To further investigate the major cellular pathways involved in DMD, we used DAVID to perform functional and pathway enrichment analysis of the 1387 DEGs (217 downregulated and 1170 upregulated). The results of these enrichment analyses (biological process, cellular component, molecular function, and KEGG pathways) are shown in Figure 3a–D. Figure 3e shows the pathway containing *CXCL12*. Cell adhesion, inflammatory response, collagen catabolic process, chemotaxis, and immune response were the most significantly enriched biological process (BP). Changes in cellular component (CC) were concentrated in the extracellular exosome, the extracellular region, the extracellular space, and the external side of the plasma membrane. With regard to molecular function (MF), receptor binding, chemoattractant activity, growth factor activity were mainly enriched. The KEGG pathway analysis showed that the chemokine signaling pathway, leukocyte transendothelial migration pathway, and NF-κB signaling pathway were the most significantly enriched.

### PPI network analysis

**Figure 4.**
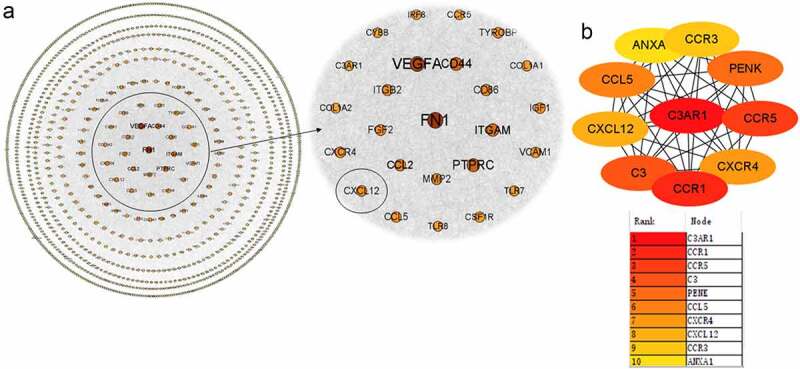
**Protein–protein interaction (PPI) network construction**. (a) The PPI network of identified DEGs, sorted by degree value. The larger the degree value, the larger count size of the node (gene). Degree value represents the quantity of edges (connections between nodes) per node. The degree value for CXCL12 is 97. (b) The 10 hub genes of the network

The STRING database was used to construct a PPI network of the 1387 EDGs. Isolated nodes were removed, and the network was visualized using Cytoscape. The completed network contained 1185 nodes (genes) and 9700 edges (interactions) (Figure 4a). The top 10 genes were identified with cytoHubba, and CXCL12 came in eighth (Figure 4b).

### Gene set enrichment analysis

**Figure 5.**
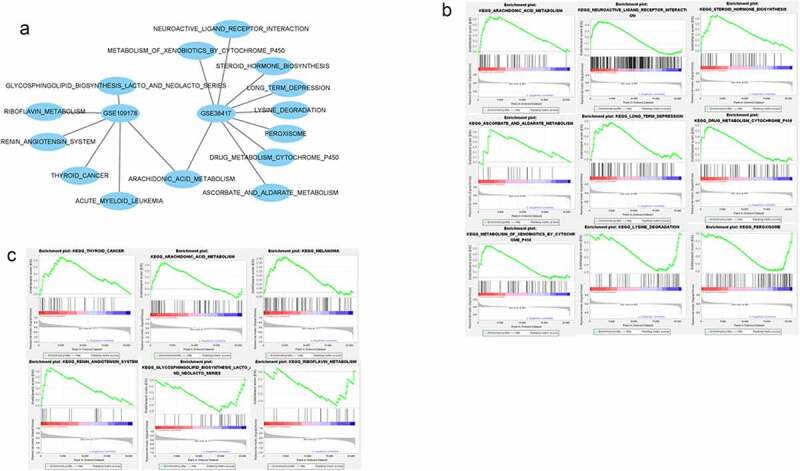
**Single-gene gene set enrichment analysis (GSEA) results for GSE38417 and GSE109178**. (a) Visualization of gene set enrichment analysis by Cytoscape. (b) Single-gene enrichment analysis of GSE38417. (c) Single-gene enrichment analysis of GSE109178

Gene set enrichment analysis showed that *CXCL12* was associated with significant enrichment of pathways involved in arachidonic acid metabolism, steroid hormone biosynthesis, peroxisome activity, lysine degradation, and inhibition of glycosphingolipid biosynthesis lacto and neolacto series (Figure 5).

## CXCL12 *and* CXCR4 *expression in mdx mice*

**Figure 6.**
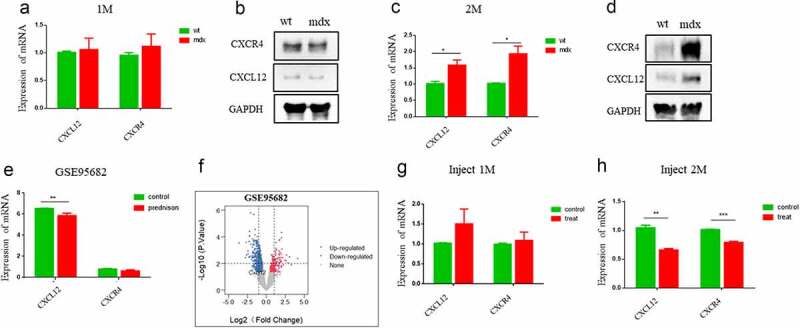
**Expression of *CXCL12* and *CXCR4* is increased in mdx mice, and metformin reduces it**. (a) Comparison of levels of *CXCL12* and *CXCR4* mRNA between mdx mice and wild-type mice at 1 month of age (n = 3 mice per group). Statistical analysis was conducted using an unpaired *t*-test. *p < 0.05. (b) Western blot comparison of CXCL12 and CXCR4 protein between mdx mice and wild-type mice at 1 month of age. (c) Comparison of levels of *CXCL12* and *CXCR4* mRNA between mdx mice and wild-type mice at 2 months of age (n = 3 mice per group). (d) Western blot comparison of CXCL12 and CXCR4 protein between mdx mice and wild-type mice at 2 months of age. (e,f) After treatment with prednisone, *CXCL12* expression declined, according to data from the GSE95682 dataset from the GEO database; |log2FoldChange| value > 0.6, *p* < 0.05. (g,h) Quantitative analysis of the relative expression level of CXCL12 and CXCR4 in untreated and metformin-treated mdx mice after 1 month and 2 months of treatment (n = 3 mice per group). Statistical analysis was conducted using an unpaired *t*-test. *p < 0.05, **p < 0.01, ***p < 0.001

The most widely used animal model of DMD is the mdx mouse. To confirm the involvement of *CXCL12* in DMD progression in model mice, we measured the levels of mRNA levels of CXCL12 and its receptor CXCR4 in mdx and WT (C57) mice at 1 and 2 months of age. Expression of *CXCL12* and *CXCR4* mRNA was similar between WT and mdx mice at 1 month but significantly upregulated in mdx mice at 2 months (Figure 6a,c). Levels of CXCL12 and CXCR4 proteins were also measured; they were not altered between WT and mdx mice at 1 month but significantly increased in mdx mice at 2 months (Figure 6b,d). Interestingly, the GSE95682 dataset showed that *CXCL12* was downregulated after prednisone treatment in mdx mice (Figure 6e,f).

Recently, metformin has been reported as a potential therapeutic agent for DMD [31–33]. In a previous study, we found that treatment with metformin improved muscle function and diminished neuromuscular deficits in mdx mice [32]. To investigate whether *CXCL12* might be involved in this improvement of muscle function, as it is with other therapeutic agents, we measured levels of *CXCL12* and *CXCR4* mRNA in mdx mice after metformin treatment. As expected, expression of both *CXCL12* and *CXCR4* was reduced after metformin treatment (Figure 6g,h). Together, these results show that *CXCL12* is upregulated in mdx mice but can be downregulated through prednisone or metformin treatment. Therefore, *CXCL12* may have potential as a novel therapeutic target for DMD treatment.

## Discussion

DMD is an X-linked recessive disease caused by mutations in the dystrophin protein [34]. Patients with DMD experience progressive proximal muscle weakness, calf hypertrophy, fibrosis, and fatty replacement of muscles [35,36]. At present, there is no fully effective treatment for DMD; glucocorticoids are the standard treatment, used to improve muscle strength and delay the onset of catastrophic respiratory and cardiac dysfunction [37]. However, long-term glucocorticoid treatment results in detrimental side effects such as short stature, obesity, pubertal delay, osteoporosis, and spine fractures [38–40]. In the present study, we aimed to identify the target genes and pathways of clinical DMD drugs (prednisone and prednisolone) by comparing the target genes of glucocorticoid drugs with the genes expressed differentially between DMD patients and normal controls. We first used the PharmGKB database to predict the target genes of glucocorticoid drugs. Next, we identified DEGs in two GSE datasets between DMD patients and healthy controls. We also performed GO biological process analysis, KEGG pathway enrichment analysis, gene set enrichment analysis, and PPI network analysis of the DEGs.

Interestingly, *CXCL12* was the only predicted glucorticoid target gene that was also a DEG in DMD patients. The CXCL12 protein is a member of the α-chemokine subfamily; it was first isolated from murine bone marrow and characterized as a pre-B-cell growth-stimulating factor. Its receptor, CXCR4, is widely expressed in different cell types throughout the body [41]. The interaction of CXCL12 with CXCR4 activates a variety of biological responses and plays a key role in numerous developmental, inflammatory, and pathological processes. The CXCL12–CXCR4 pathway is reported to be downregulated in skeletal muscles in patients with cancer-associated cachexia, and activation of this pathway protects muscles from wasting in mice with this syndrome [42]. In addition, mobilization of CXCR4 expressing satellite cells has been shown to depend on CXCL12 signaling, and thus the CXCL12–CXCR4 axis plays a pivotal role in skeletal muscle regeneration [43–45].

Muscle inflammation, that caused by severe damage and cycles of muscle fiber necrosis and regeneration, is one of the main pathological features of DMD [46]. This muscle inflammatory response is regulated in a complex way through the interaction of soluble factors and adhesion molecules [47]. Chemotactic cytokines play a key role in activation and directed migration of inflammatory cells to the tissues [48,49]. CXCL12, a member of chemotactic cytokine, was reported be induced in DMD patients and DMD model mdx mice [50–52]*****. Blocking or reducing of individual cytokines and chemokines including CXCL12 may be a feasible method for the treatment of DMD and an amenable approach to reduce side effects of anti-inflammatory therapy. However, on the other hand, In DMD patients, muscle injury and atrophy lead to abnormal blood flow and functional ischemia, and chemokine CXCL12α can assist endothelial circulating progenitor cells in mediating vascular and muscle repair [53,54]. In mdx mice, CXCR4 was also found to tightly control muscle development and regeneration [55]. Abundant evidence suggests increased levels of CXCL12 and CXCR4 expression in satellite cells can promote muscle regeneration [43,44,56], and CXCR4 can enhance engraftment of muscle progenitor cells [57]. In DMD patients, this process may not balance the process of muscle inflammation caused by degenerative disease. Therefore, use of CXCL12/CXCR4 as a target for anti-inflammatory therapy requires careful dose control [58,59].

In this study, KEGG and GO analyses of DEGs, including *CXCL12*, showed enrichment of inflammation-related responses, cell adhesion, and response to hypoxia. In DMD patients, mutated dystrophin triggers chronic inflammation, leading to migration of immunocytes and chemokines will migrate [60–64]; chemotaxis and migration capacity of polymorphonuclear leukocytes are significantly reduced in DMD patients aged 2–14 years [65]. Meanwhile, NF-κB response is increased in DMD, promoting inflammation and impairing muscle regeneration [66,67]. In mdx mice, integrins and pro-inflammatory cytokines are upregulated, indicating altered immune regulation [68]. Thus, inhibiting inflammation may serve as a therapeutic strategy for DMD.

The dystrophin–glycoprotein complex, composed of dystrophin and many other proteins, is an important component in maintaining cell adhesion [69], and mutant dystrophin is the cause of DMD; thus, weakened cell adhesion plays an important role in DMD [70]. Due to degeneration of the diaphragm, DMD is usually accompanied by respiratory weakness [71,72], which may subject the cells in the body to a state of hypoxia. It has been reported that the *Drosophila* model of DMD (*dmDys*) is more sensitive to hypoxia-induced muscle dysfunction, suggesting that targeting hypoxia may have potential as a novel therapeutic strategy in DMD [73]

The mdx mouse is a widely used model of DMD [2,74]. Our results showed that mRNA and protein levels of *CXCL12* and *CXCR4* were increased in mdx mice at 2 months of age, which may be caused by muscle inflammation [75]. After treatment with prednisone, the expression of *CXCL12* declined. This result is consistent with the DMD patient data from the two GSE datasets. Metformin has been suggested as a potential treatment for DMD, with the advantages of low price and few side effects [31–33]. Our results showed that levels of *CXCL12* and *CXCR4* mRNA were reduced after 2 months of metformin treatment in mdx mice, with skeletal muscle function showing significant improvement [32]. We hypothesized that downregulation of *CXCL12* and *CXCR4* is a secondary effect of metformin treatment in mdx mice.

## Conclusion

In conclusion, we aimed to identify a target gene or pathway affected by glucocorticoid treatment in DMD, which might provide target ideas for novel drugs. *CXCL12* was identified as a glucocorticoid target gene that was differentially expressed in DMD patients. In terms of functional enrichment and pathway enrichment, *CXCL12* mainly affected the inflammatory response and muscle metabolism. We also found that expression of CXCL12 and its receptor CXCR4 was elevated in mdx mice, but decreased after treatment with prednisone. This result is consistent with the expression profiles of human DMD patients obtained from GEO datasets. However, further studies are required to elucidate the biological functions of these proteins.

## Figures and Tables

**Table:** 

*CXCL12*	C-X-C motif chemokine ligand 12
DMD	Duchenne muscular dystrophy
DEGs	differentially expressed genes
PharmGKB	Pharmacogenetics and Pharmacogenomics Knowledge Base
KEGG	Kyoto Encyclopedia of Genes and Genomes
GO	Gene Ontology
GSEA	Gene Set Enrichment Analysis
PPI	protein–protein interaction
STRING	Search Tool for the Retrieval of Interacting Genes
*CXCR4*	C-X-C motif chemokine receptor 4
Mdx	X-linked muscular dystrophy
GEO	Gene Expression Omnibus

## Data Availability

The datasets were acquired from the National Center for Biotechnology Information Gene Expression Omnibus (GEO) database (https://www.ncbi.nlm.nih.gov/) and the Pharmacogenetics and Pharmacogenomics Knowledge Base (PharmGKB) database (https://www.pharmgkb.org).

## Highlights

1. Differentially expressed genes (DEGs) between DMD patients and healthy human controls were identified and analyzed.
2. *CXCL12* is the only one of these DEGs that is also a predicted glucocorticoid target gene.
3. *CXCL12* and its receptor CXCR4 showed increased expression in DMD mouse models (mdx mice).
